# Construction and External Validation of a Ferroptosis-Related Gene Signature of Predictive Value for the Overall Survival in Bladder Cancer

**DOI:** 10.3389/fmolb.2021.675651

**Published:** 2021-05-21

**Authors:** Jingchao Liu, Hong Ma, Lingfeng Meng, Xiaodong Liu, Zhengtong Lv, Yaoguang Zhang, Jianye Wang

**Affiliations:** ^1^ Department of Urology, Beijing Hospital, National Center of Gerontology, Institute of Geriatric Medicine, Chinese Academy of Medical Sciences, Beijing, China; ^2^ Graduate School of Peking Union Medical College, Chinese Academy of Medical Sciences, Beijing, China

**Keywords:** ferroptosis, GEO, TCGA, bladder cancer, gene signature

## Abstract

**Purpose:** To identify whether ferroptosis-related genes play predictive roles in bladder cancer patients and to develop a ferroptosis-related gene signature to predict overall survival outcomes.

**Materials and Methods:** We downloaded the mRNA expression files and clinical data of 256 bladder samples (188 bladder tumour and 68 nontumour samples) from the GEO database and 430 bladder samples (411 bladder tumour and 19 nontumour samples) from the TCGA database. A multigene signature based on prognostic ferroptosis-related genes was constructed by least absolute shrinkage and selection operator Cox regression analysis in the GEO cohort. The TCGA cohort was used to validate the ferroptosis-related gene signature. Next, functional enrichment analysis, including both Kyoto Encyclopedia of Genes and Genomes and Gene Ontology analyses, was performed to elucidate the mechanism underlying the signature. The ssGSEA scores of 16 immune cells and 13 immune-related pathway activities between the high-risk and low-risk groups were also analysed in our study.

**Results:** Thirty-three (67.3%) ferroptosis-related genes were differentially expressed between bladder tumour samples and nontumour samples in the GEO cohort. The intersection of prognostic ferroptosis-related genes and differentially expressed genes identified four prognostic targets, including ALOX5, FANCD2, HMGCR and FADS2. The least absolute shrinkage and selection operator Cox regression successfully built a 4-gene signature: risk score value = e^sum^ (each gene’s normalized expression * each gene’s coefficient). Univariate and multivariate Cox regression analyses were performed in both the GEO and TCGA cohorts to test the independent prognostic value of the 4-gene risk signature. Multivariate Cox regression analysis in the GEO cohort identified age (*p* < 0.001), grade (*p* = 0.129) and risk score (*p* = 0.016) as independent prognostic predictors for overall survival. Multivariate Cox regression analysis in the TCGA cohort also identified age (*p* = 0.002), stage (*p* < 0.001) and risk score (*p* = 0.006) as independent prognostic predictors for overall survival. The type II IFN response was determined to be significantly weakened in the high-risk group in both the GEO and TCGA cohorts.

**Conclusion:** We successfully built a ferroptosis-related gene signature of significant predictive value for bladder cancer. These results suggest a novel research direction for targeted therapy of bladder cancer in the future.

## Introduction

Bladder cancer is one of the most prevalent cancers originating from the urinary tract and causes high levels of morbidity and mortality worldwide. Approximately 75% of bladder cancer cases are classified as nonmuscle invasive bladder cancer (NMIBC), and 25% are classified as muscle invasive bladder cancer (MIBC) (Dy et al., 2017). Although NMIBC can be treated by surgery, it often relapses further after resection, and approximately 15% of cases deteriorate to MIBC, which is related to more aggressive characteristics and poor prognosis (Witjes et al., 2021). The traditional pathological grade and clinical TNM stage are currently utilized to evaluate the prognosis of bladder cancer patients. Some studies have reported that the mutation of tumour suppressor genes to oncogenes, such as HER-2, H-Ras and Bcl-2, promotes the occurrence of bladder cancer (Montie, 2005). The inactivation of suppressor genes encoding proteins regulating DNA repair or apoptosis may also be one possible mechanism (Williams, 2004). Various immune-related pathways are also reported to contribute to the occurrence of bladder cancer (Wu et al., 2020; Yan et al., 2020).

Ferroptosis, a completely new form of regulated cell death, was first reported in 2012 (Dixon et al., 2012). In the past several years, researchers worldwide have had great interest in studying ferroptosis (Chen et al., 2021). Ferroptosis is an iron-dependent mode of programmed cell death characterized by the accumulation of lipid peroxides. Although ferroptosis is a recently named concept, it is firmly believed to be an ancient and widespread form of cell death, both in mammalian systems and evolutionarily remote species (Bogacz and Krauth-siegel, 2018). Notably, one type of oxidative regulated cell death named oxytosis in 2001 was recently believed to be one manifestation of ferroptosis (Tan et al., 2001). Various cutting edge research studies have illustrated that ferroptosis plays a vital role in the mechanism of many cancers, including hepatocellular carcinoma, colorectal cancer, breast cancer, non-small-cell lung cancer and acute myeloid leukemia (Ryu et al., 2011; Louandre et al., 2013; Guo et al., 2018). Cytotoxic drugs or targeted drugs have been widely used in the clinic, but a large proportion of cancer patients show obvious drug resistance. Increasing evidence suggests that many regulators of ferroptosis can benefit cancer patients who are resistant to conventional drugs and are more likely to metastasize (Viswanathan et al., 2017; Tsoi et al., 2018). The induction of ferroptosis has become a promising research direction in the treatment of cancer. Integrated genetic information has been reported to help distinguish tumours that are sensitive to ferroptosis induction treatment (Bailey et al., 2018). To our knowledge, there is no research studying the relationship between bladder cancer and ferroptosis. We conducted the present study to identify whether ferroptosis-related genes play a predictive role in bladder cancer patients and to build a ferroptosis-related gene signature to predict overall survival outcomes. We identified four ferroptosis-related genes of predictive value, constructed a 4-gene ferroptosis signature with the Gene Expression Omnibus (GEO) cohort and validated it with The Cancer Genome Atlas (TCGA) cohort. Furthermore, we also performed relative functional analysis to illustrate the possible mechanism of ferroptosis in bladder cancer.

## Materials and Methods

### Data Collection and Ferroptosis-Related Gene Acquisition

We downloaded the mRNA expression files and clinical data of 256 bladder samples from the GEO database (GSE13507) (Kim et al., 2010). All 256 samples were used to analyse the differentially expressed genes (DEGs) between the tumour and nontumour samples. Only 165 tumour samples with corresponding clinical information were used to perform univariate Cox regression. The mRNA expression data and clinical documents of 430 bladder tumour patients were also downloaded from the TCGA database up to January 2021. Only 400 bladder tumour patients from the TCGA cohort, including corresponding clinical data and information on the four target genes, were included in the validation analysis. The “limma” packages in R software were utilized to normalize the gene expression profiles of both the GEO and TCGA samples. Because all data in our analysis are available to the public in the GEO and TCGA databases, ethical approval of the local institution was not needed for this study. All processes in the present study followed the relevant guidelines and policies of the GEO and TCGA databases.

We reviewed previous studies on the topic of ferroptosis published in the last 3 years to identify all reported ferroptosis-related genes. All searched high-quality studies on ferroptosis were defined as impact factors > 10 points, and 60 ferroptosis-related genes were identified in total (Stockwell et al., 2017; Bersuker et al., 2019; Doll et al., 2019; Hassannia et al., 2019). Supplementary Table S1 lists all the ferroptosis-related genes. In total, 54 ferroptosis-related genes were identified in the gene-matrix probe from GSE13507 tumour samples, and 49 ferroptosis-related genes were identified in both the tumour samples and nontumour samples in GSE13507.

### Development and External Validation of a Ferroptosis-Related Gene Signature

The DEGs between bladder tumour samples and nontumour samples in the GEO database were identified by the “limma” packages in R software (*p* < 0.05). The prognostic ferroptosis-related genes were identified by univariate Cox regression analysis. Benjamini and Hochberg (BH) correction was utilized to adjust the *p* value. The STRING database was adopted to illustrate a network of the overlapping prognostic DEGs (Szklarczyk et al., 2011). Based on the results of DEG analysis and univariate Cox regression, we developed a prognostic gene model by least absolute shrinkage and selection operator (LASSO) Cox regression analysis (Simon et al., 2011). The “glmnet” package in R software was utilized for LASSO Cox regression to select and delete relevant genes. Normalized expression data of prognostic DEGs in the GEO cohort were viewed as independent variables, and the corresponding overall survival time and patient survival states (dead or live) were viewed as response variables. Tenfold cross-validation following the minimum criteria was used to determine the penalty parameter (*λ*) in our prognostic model. Each gene’s normalized expression value and its LASSO Cox regression coefficients were combined to calculate every patient’s risk score using the following specific calculation formula: risk score value = e^sum (each gene^’^s normalized expression * each gene^’^s coefficient)^. The risk score indicated the expression level of target ferroptosis-related genes in the present study. The results of the calculation formula indicated patients’ risk scores. The median results of the risk score were utilized to stratify each cohort into a high-risk group and a low-risk group. The “prcomp” function of the “Stats” packages in R software was used to perform principal component analysis (PCA). Moreover, the “Rtsne” package in R software was also used to perform the t-SNE analysis to study the distribution pattern of different risk groups. The correlation between the present risk scores and published bladder molecular subtypes was explored by the “limma,” “ggplot2” and “ggpubr” packages in R software (Robertson et al., 2017). The correlation between the present risk scores and known major molecular alterations (TP53, FGFR3, RB1, ATM, ERBB2 and DNA repair like ERCC2) was also evaluated in the present study. The “surv-cutpoint” function in the “survminer” package in R software was used to determine the optimal cut-off value during survival analysis. The independent prognostic value for survival of the risk score system was then validated by univariate and multivariate Cox regression analysis in both the GEO and TCGA cohorts.

### Functional Enrichment Analysis

The DEGs between the high-risk group and low-risk group were analysed in both the GEO and TCGA cohorts. Then, functional enrichment analysis, including both Kyoto Encyclopedia of Genes and Genomes (KEGG) and Gene Ontology (GO) analyses, was performed by the “clusterProfiler” package in R software. The BH method was utilized to adjust the *p* value. Single-sample gene set enrichment analysis (ssGSEA) was used to calculate the infiltration score of 16 immune cells and 13 immune-related pathways by the “gsva” package in R software (Rooney et al., 2015). The related reference gene file is supplied in Supplementary Table S2.

### Statistical Analysis

The DEGs between bladder tumour samples and nontumour samples were identified by Student’s t-test. The Chi-squared test was used to compare the categorical variables in the present study. The ssGSEA scores of 16 immune cells and 13 immune-related pathway activities between the high-risk and low-risk groups were compared by the Mann-Whitney test, with an adjusted *p* value using the BH method. Kaplan–Meier analysis by the log-rank test was utilized to compare survival data between different risk groups. Univariate and multivariate Cox regression analyses were performed to identify independent risk factors for survival status in both the GEO and TCGA cohorts. All analysis procedures were performed using either R software (V 3.5.3) or SPSS (V 23.0). If not specified, a significant *p* value was defined as <0.05, and all values were two-tailed.

## Results

### Patient Demographics and Characteristics

**FIGURE 1:**
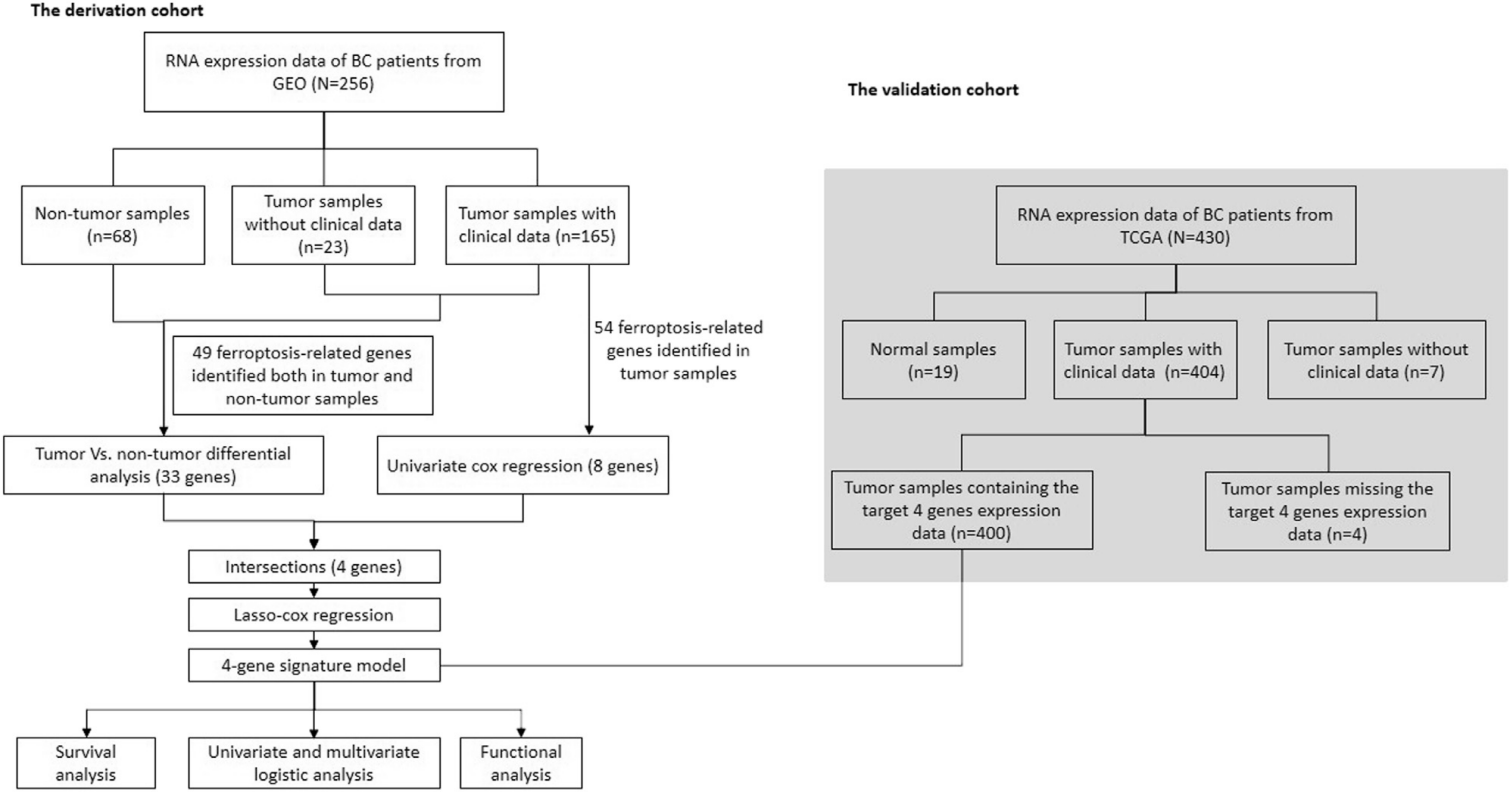
Flow chart for our data selection and analysis.

**FIGURE 2:**
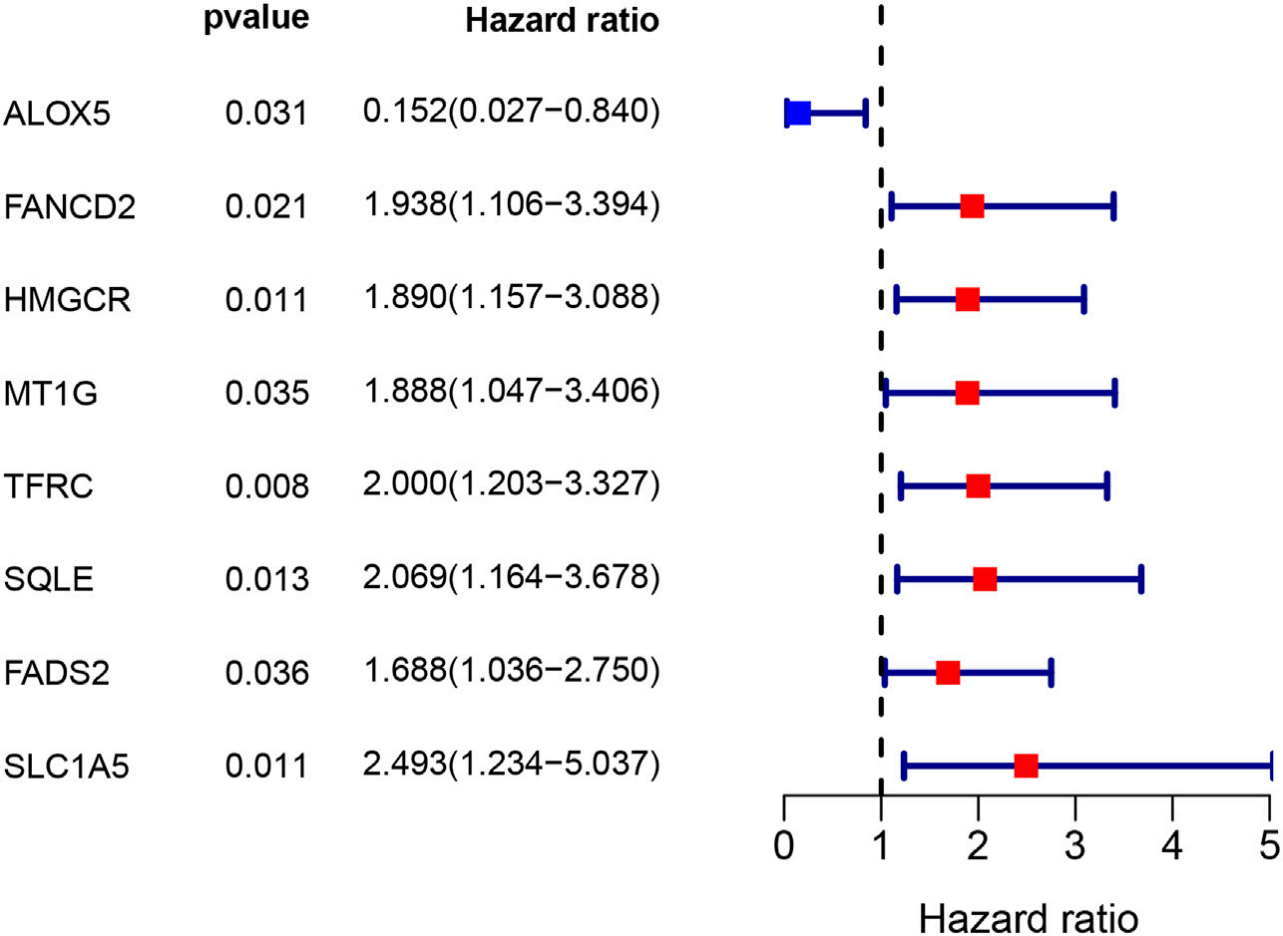
Eight ferroptosis-related genes of predictive value for overall survival.

Figures 1, 2 illustrates the flow chart of the present study. The mRNA transcriptome expression data of 256 samples from the GEO database and 430 samples from the TCGA database were downloaded in the first step. The GEO cohort was designed as the trial cohort, and the TCGA cohort was designed as the validation cohort. For the GEO cohort, there were 68 nontumour samples, 23 tumour samples without corresponding clinical information, and 165 tumour samples with available clinical information. Notably, the downloaded tumour sample probes and nontumour probes of the GEO cohort were different, and 49 (81.7%) ferroptosis-related genes identified in both tumour samples and nontumour samples were included in the DEG analysis. In total, 54 (90%) ferroptosis-related genes were identified in 165 bladder tumour sample probes and were further included in the univariate Cox regression to select prognostic ferroptosis-related genes. For the TCGA cohort, there were 19 nontumour samples, 404 tumour samples with available clinical data and seven tumour samples without corresponding clinical data. Considering the gene probe matrix for the TCGA cohort used in the present study, 400 bladder tumour samples with clinical information presented target genes, and these samples were further utilized to validate the present signature model. All detailed patient characteristics are summarized in Table 1.

**TABLE 1:** Characteristics of BC patients used in this study.

	GEO cohort	TCGA cohort
No	165	400
Age (median, range)	65.2 (24–88)	67.9 (34–89)
Gender (%)		
Female	30 (18.2%)	103 (25.8%)
Male	135 (81.8%)	297 (74.3%)
Grade (%)		
Low grade	105 (63.6%)	20 (5.0%)
High grade	60 (36.4%)	377 (94.3%)
Unknown	0	3 (0.8%)
Stage (%)		
Ⅰ	NA	2 (0.5%)
Ⅱ	NA	127 (31.8%)
Ⅲ	NA	138 (34.5%)
Ⅳ	NA	131 (32.8%)
Unknown	0	2 (0.5%)
OS months (median, range)	48.38 (1.03–136.97)	26.98 (0.43–168.33)
Status (%)		
Alive	96 (58.2%)	225 (56.2%)
Dead	69 (41.8%)	175 (43.8%)
Prognostic Gene		
ALOX5	3.61 (3.13–3.85)	4.10 (0.33–7.29)
FANCD2	3.28 (2.99–3.53)	2.17 (0.474.22)
HMGCR	3.58 (3.43–3.78)	3.23 (1.53–5.45)
FADS2	3.15 (3.00–3.45)	2.49 (0.17–6.23)
Risk score (median, range)	14.03 (13.12–15.24)	9.09 (−4.74 to 24.68)
Risk group (%)		
Low	83 (50.3%)	340 (85%)
High	82 (49.7%)	60 (15%)

GEO, gene expression omnibus; TCGA, the cancer genome atlas; OS, overall survival; NA, not available.

### Identification of Differentially Expressed Genes and Prognostic Ferroptosis-Related Genes in the Gene Expression Omnibus Cohort

**FIGURE 3:**
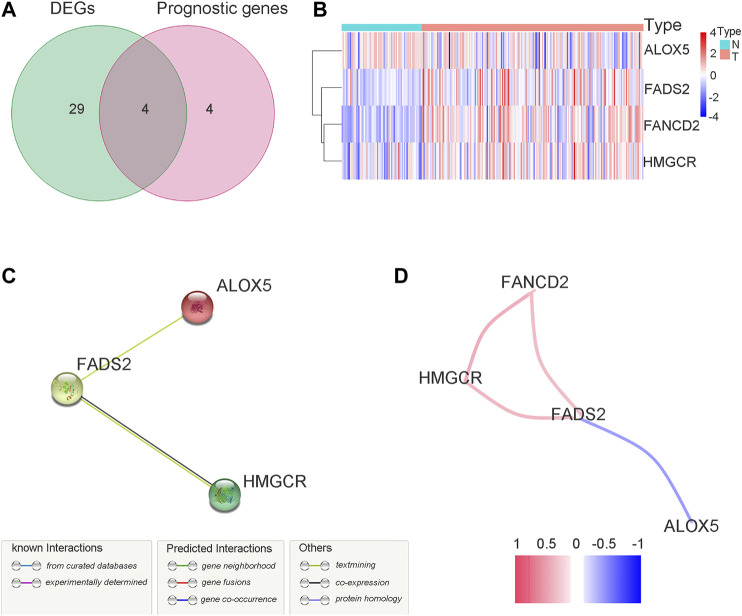
Identification of the target ferroptosis-related genes during GEO cohort. **(A)** Venn diagram to identify prognostic DEGs in GEO cohort. **(B)** The expression pattern of candidate ferroptosis-related genes by heatmap. **(C)** The interactions between candidate genes with PPI analysis. **(D)** Connection network for candidate ferrotosis-related genes. Colors are utilized to present different correlation coefficients.

A total of 33 (67.3%) ferroptosis-related genes were significantly different between the bladder tumour samples and nontumour samples in the DEG analysis for the GEO cohort. The differences in ferroptosis-related genes and detailed DEG expression files are shown in Supplementary Table S3, Supplementary Table S4. During the univariate Cox regression analysis, only eight ferroptosis-related genes were found to be prognostic for patients. The intersection of prognostic ferroptosis-related genes and DEGs identified four prognostic DEGs, including ALOX5, FANCD2, HMGCR and FADS2. The detailed expression pattern of the four genes between tumour and nontumour samples is shown by the heatmap in Figure 3B. Furthermore, the interaction network and expression correlation among the four genes are shown in Figures 3C,D. The correlation analysis between the significant genes is also illustrated in Supplementary Table S5.

### Development and External Validation of a Ferroptosis-Related Gene Signature

The above four genes were then analysed by LASSO Cox regression analysis to build a prognostic model. The LASSO Cox regression successfully identified a 4-gene signature, and the results are listed in Table 2. Univariate Cox analysis indicated that high expression of ALOX5 was correlated with better prognosis and that high expression of FANCD2, HMGCR and FADS2 was correlated with poor prognosis (Supplementary Table S4). The specific calculation formula is as follows: risk score value = e^(1.882* expression value of FANCD2+2.661* expression value of HMGCR+1.573* expression value of FADS2–1.835* expression value of ALOX5).^ All samples were then divided into a high-risk group or a low-risk group depending on the median cut-off value. (Supplementary Table S6). A total of 82 patients were stratified into the high-risk group, and 83 patients were stratified into the low-risk group in the GEO cohort. Depending on the abovementioned calculation formula, the risk scores of 400 bladder tumour samples from the TCGA cohort were also calculated, and the risk group distributions are shown in Supplementary Table S7. The complete clinical information of the trial group (Supplementary Table S8) and validation group (Supplementary Table S9) used in the present study are presented in the supplementary files.

**TABLE 2:** Results of Lasso–Cox regression analysis.

Gene	Coefficient
ALOX5	−1.835
FANCD2	1.882
HMGCR	2.661
FADS2	1.573

Lasso–Cox, least absolute shrinkage and selection operator Cox regression analysis.

Patient characteristics in different risk groups for both the GEO and TCGA cohorts are listed in Table 3. In the GEO cohort, the high-risk group was found to have a significant correlation with higher tumour grade (*p* < 0.01) and older age (*p* = 0.05). In the TCGA cohort, the high-risk group was also found to be significantly correlated with higher tumour grade (*p* = 0.027).

**TABLE 3:** Patient characteristics in different risk groups.

	GEO	Cohort		TCGA	Cohort	
	High risk	Low risk	*p*	High risk	Low risk	*p*
Gender			0.212			0.269
Female	18 (60%)	12 (40%)		12 (11.7%)	91 (88.3%)	
Male	64 (47.4%)	71 (52.6%)		48 (16.2%)	249 (83.8%)	
Grade			**<0.01**			**0.027**
Low	37 (35.2%)	68 (64.8%)		0	20	
High	45 (75%)	15 (25%)		59	318	
Unknown	0	0		1	2	
Age (year)			0.050			0.808
≤65	30 (47%)	44 (53%)		23 (14.5%)	136 (85.5%)	
>65	52 (63.4%)	39 (36.6%)		37 (15.4%)	204 (84.6%)	
Catagory			**<0.01**			**<0.01**
NMIBC	41 (39.8%)	62 (60.2%)		1 (25%)	3 (75%)	
MIBC	41 (66.1%)	21 (33.9%)		59 (14.9%)	337 (85.1%)	
Stage						0.464
Ⅰ+Ⅱ	–	–		17 (13.2%)	112 (86.8%)	
Ⅲ+Ⅳ	–	–		43 (16%)	226 (84%)	
Unknown				0	2 (100%)	

Bold values indicate statistically significant (p<0.05).

**FIGURE 4:**
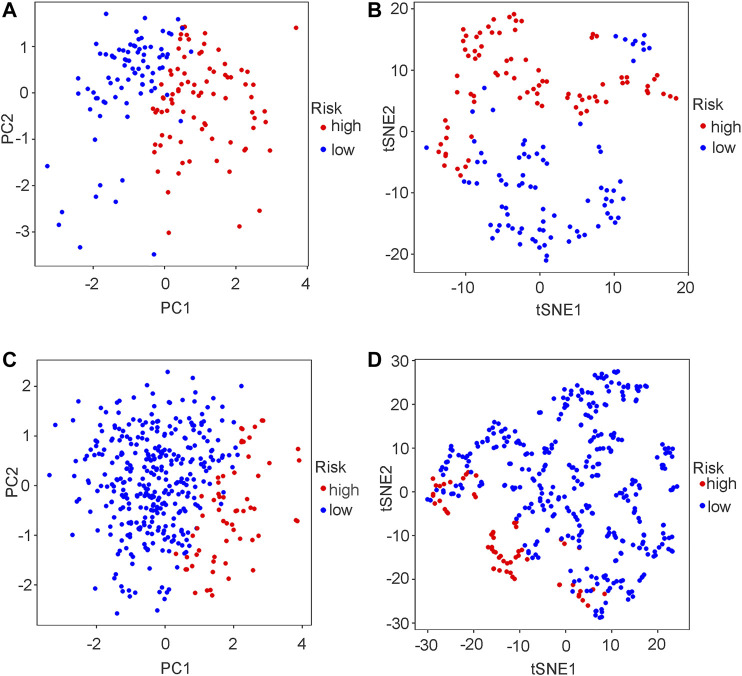
PCA plot and t-SNE analysis in both GEO and TCGA cohort. **(A)** PCA plot in GEO cohort. **(B)** t-SNE analysis in GEO cohort. **(C)** PCA plot in TCGA cohort. **(D)** t-SNE analysis in TCGA cohort.

**FIGURE 5:**
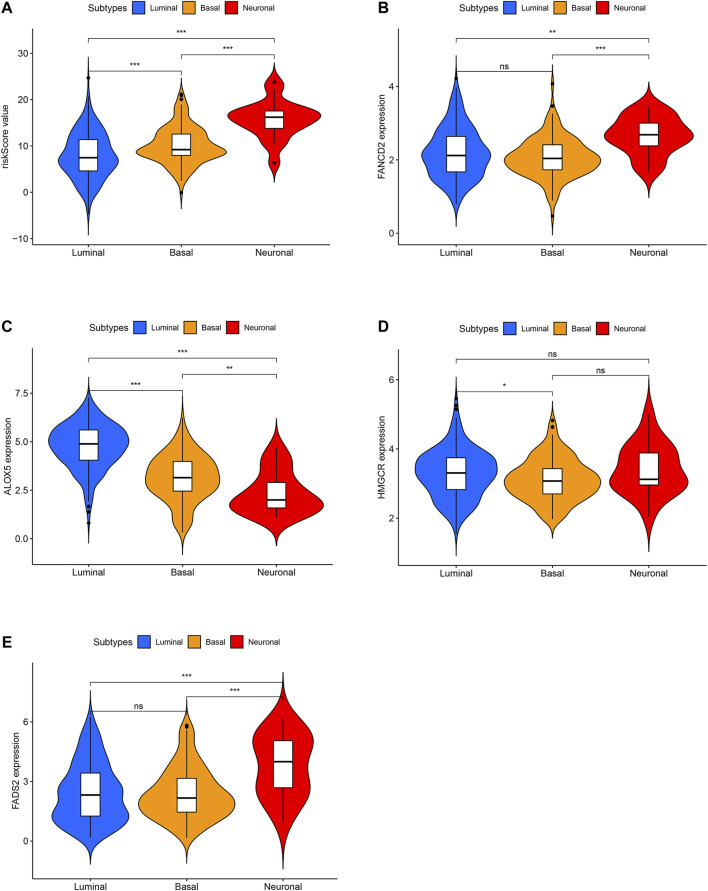
The difference analysis for risk scores between different molecular subtypes in TCGA cohort. **(A)** The difference analysis for risk scores between different molecular subtypes. **(B)** The difference analysis for FANCD2 expression between different molecular subtypes. **(C)** The difference analysis for ALOX5 expression between different molecular subtypes. **(D)** The difference analysis for HMGCR expression between different molecular subtypes. **(E)** The difference analysis for FADS2 expression between different molecular subtypes.

**FIGURE 6:**
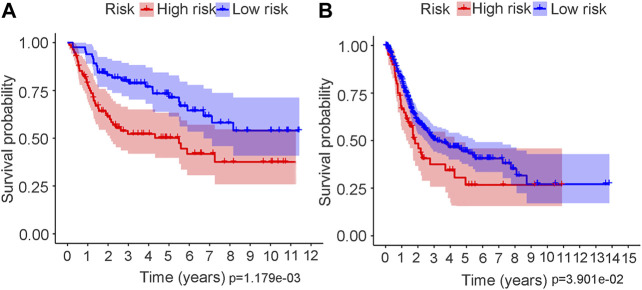
Kaplan–Meier survival analysis in both GEO and TCGA cohort. **(A)** Kaplan–Meier curve in GEO cohort. **(B)** Kaplan–Meier curve in TCGA cohort.

The risk score distributions of the GEO cohort (Supplementary Figure S1A) and the TCGA cohort (Supplementary Figure S1B) are illustrated in Supplementary Figure S1. Supplementary Figure S1C shows that the high-risk group in the GEO cohort had a higher proportion of deaths than the low-risk group, which was confirmed in the TCGA cohort (Supplementary Figure S1D). Figure 4 shows the PCA and t-SNE analysis in both the GEO and TCGA cohorts. Figures 4A,B indicate that the patients with different risk groups in the GEO cohort were distributed in different directions. Figures 4C,D indicate that the patients with different risk groups in the TCGA cohort were distributed in two different directions. The two risk groups barely intersected in both PCA and t-SNE analysis, indicating feasibility for the usage of the above 4-gene signature. The molecular subtypes (Basal and Luminal) are one of the most important findings in recent years. Molecular subtype information is available for the TCGA cohort (Robertson et al., 2017). The corresponding data for the present cohort are shown in Supplementary Table S10. We identified that the “Basal” subtypes tended to obtain higher risk scores than the “Luminal” subtypes. (*p* < 0.01) The “Neuronal” subtypes also obtained significantly higher risk scores than the “Basal” subtypes. (Figure 5). The correlation analysis between risk scores and major known molecular alterations also identified a significant relationship between the risk scores and TP53 alterations. (Supplementary Table S11, Supplementary Table S12). Furthermore, Kaplan–Meier curves were further analysed in both the GEO and TCGA cohorts. Kaplan–Meier curves illustrated that the high-risk group had a worse survival outcome than the low-risk group in the GEO cohort (*p* = 0.01, Figure 6A). Consistently, a significantly worse survival outcome for the high-risk group was also identified in the TCGA cohort (*p* = 0.039, Figure 6B).

### Independent Prognostic Value of the Signature in Trial and Validation Cohorts

**FIGURE 7:**
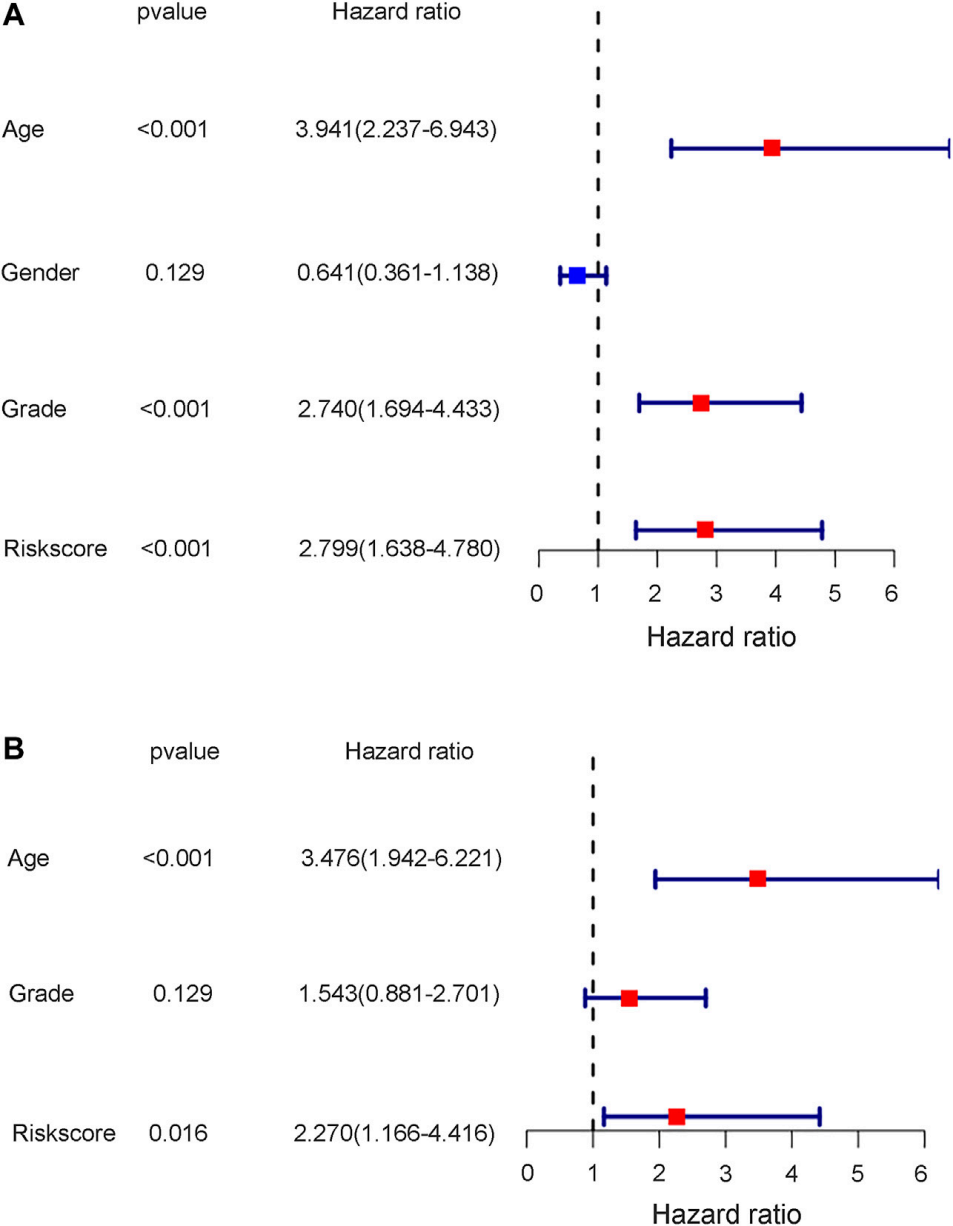
The results of univariate and multivariate cox regression analysis in GEO cohort. **(A)** The univariate cox regression analysis in GEO cohort. **(B)** The multivariate cox regression analysis in GEO cohort.

**FIGURE 8:**
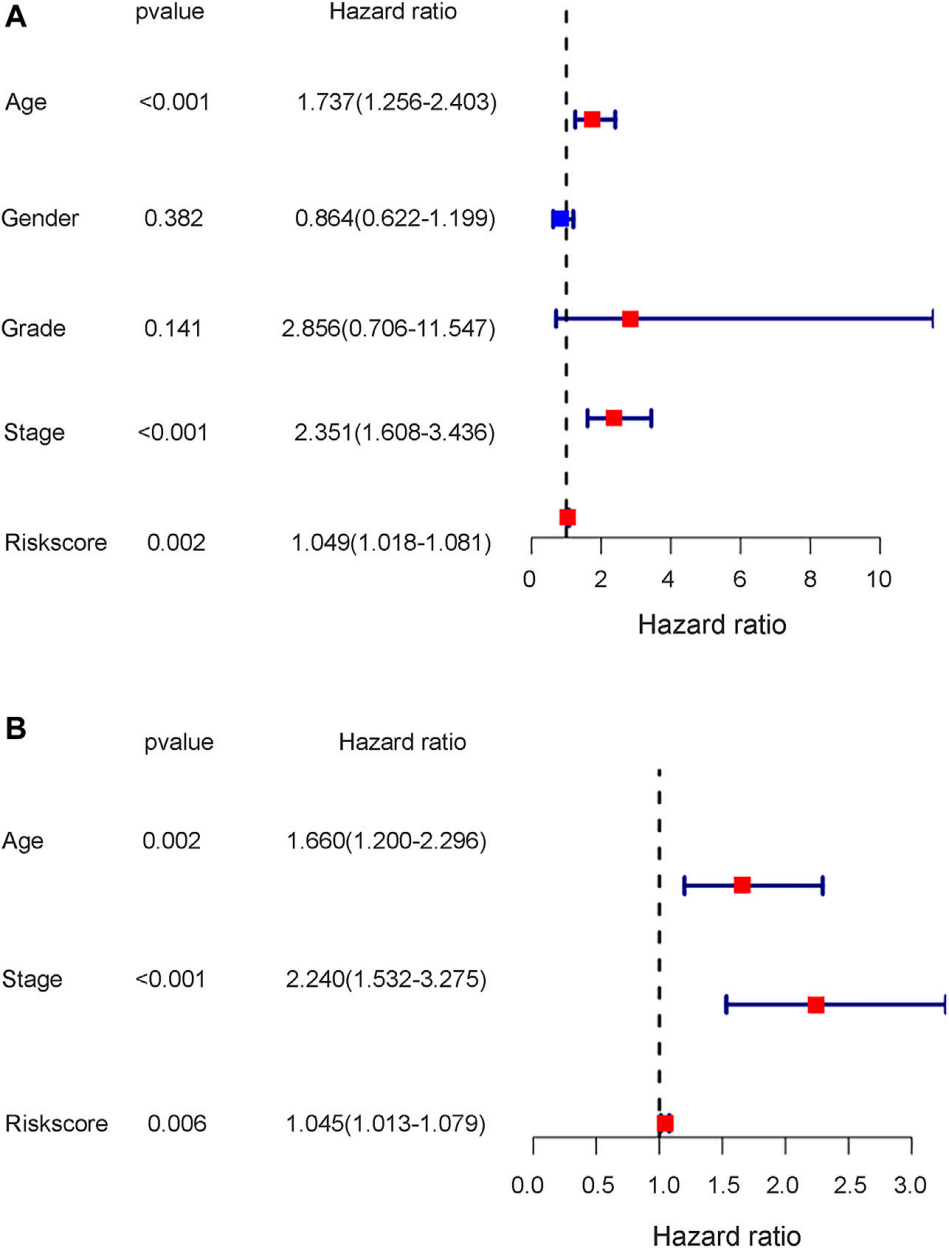
The results of univariate and multivariate cox regression analysis in TCGA cohort. **(A)** The univariate cox regression analysis in TCGA cohort. **(B)** The multivariate cox regression analysis in TCGA cohort.

To further test the independent prognostic value of the 4-gene ferroptosis signature, univariate and multivariate Cox regression analyses were performed in both the GEO and TCGA cohorts. For the GEO cohort, univariate Cox regression analysis identified age (*p* < 0.001), grade (*p* < 0.001) and risk score (*p* < 0.001) as prognostic factors for OS (Figure 7A). Further multivariate Cox regression analysis identified only age (*p* < 0.001) and risk score (*p* = 0.016) as independent prognostic predictors for OS (Figure 7B). Notably, for the TCGA cohort, univariate Cox regression analysis identified age (*p* < 0.001), stage (*p* < 0.001) and risk score (*p* = 0.002) as prognostic factors for OS (Figure 8A). Further multivariate Cox regression analysis also identified age (*p* = 0.002), stage (*p* < 0.001) and risk score (*p* = 0.006) as independent prognostic predictors for OS (Figure 8B). Furthermore, we also included four target genes, our risk scores and other clinical data in multivariate analysis in both the trial and validation cohorts. Consistent with the above analysis, only age (*p* < 0.001) and risk score (*p* < 0.001) were identified as independent prognostic variables in the GEO cohort. (Supplementary Table S13). For the TCGA cohort, age (*p* < 0.001), stage (*p* < 0.001), risk score (*p* = 0.01) and FANCD2 were identified as independent prognostic variables. (Supplementary Table S14).

### Functional Analyses in the Trial and Validation Cohorts

**FIGURE 9:**
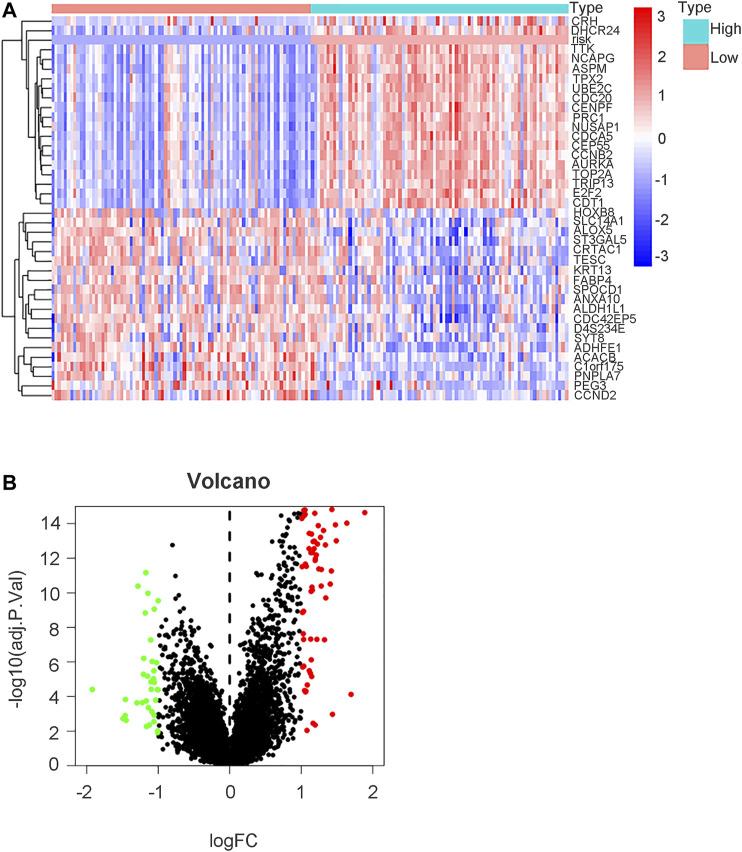
DEGs analysis between high risk group and low risk group in GEO cohort. **(A)** Gene heatmap showing distribution of DEGs between high risk group and low risk group in GEO cohort. The term “risk” indicated our risk signature and other terms indicated DEGs in our analysis. **(B)** Volcano map of DEGs between high risk group and low risk group (|logFC| > 1, P. adjust < 0.05).

**FIGURE 10:**
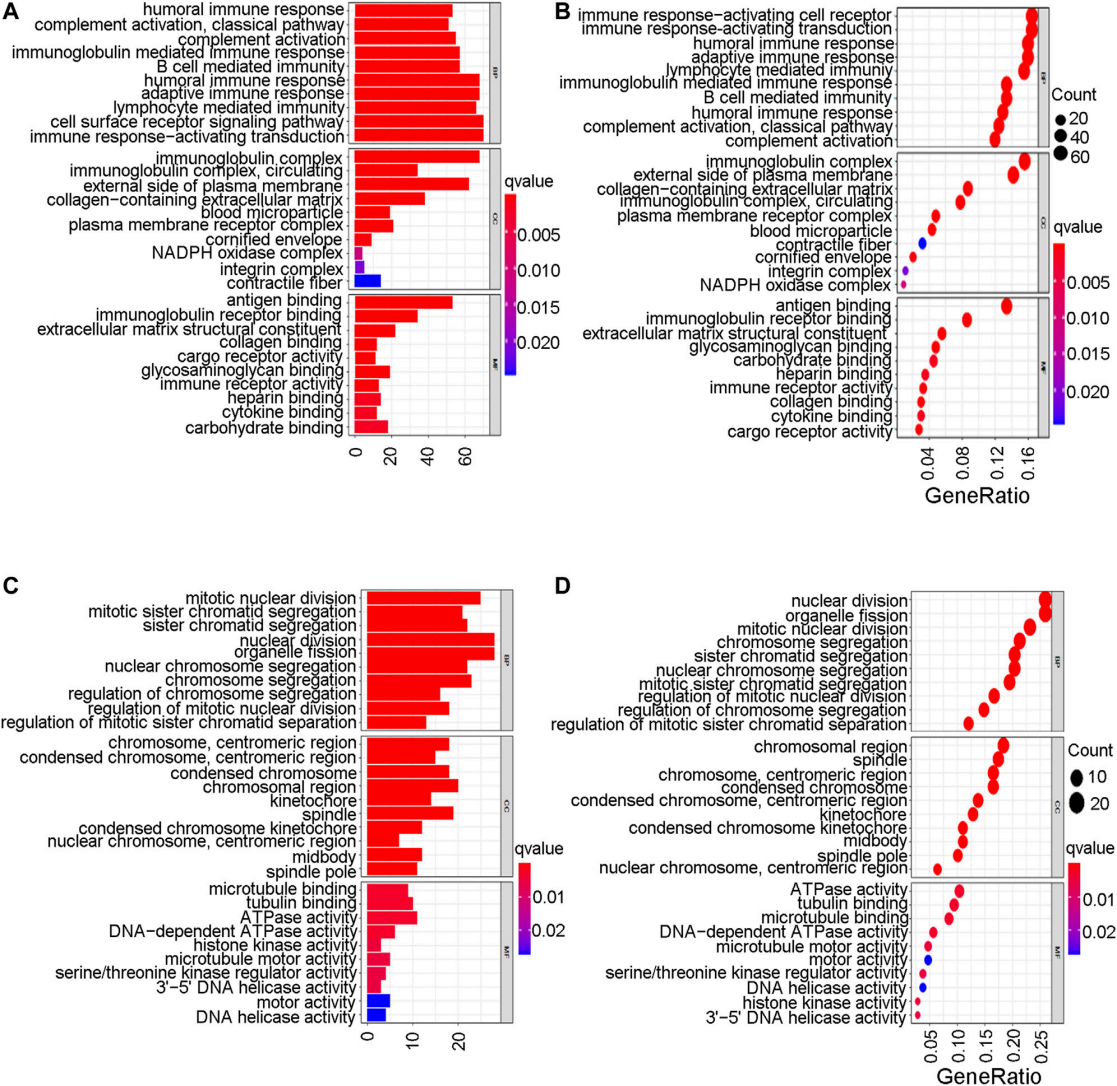
Go enrichment analysis. **(A)** The GO enrichment analysis by barplot in TCGA cohort. **(B)** The GO enrichment analysis by bubble chart in TCGA cohort. **(C)** The GO enrichment analysis by barplot in GEO cohort. **(D)** The GO enrichment analysis by bubble chart in GEO cohort.

**FIGURE 11:**
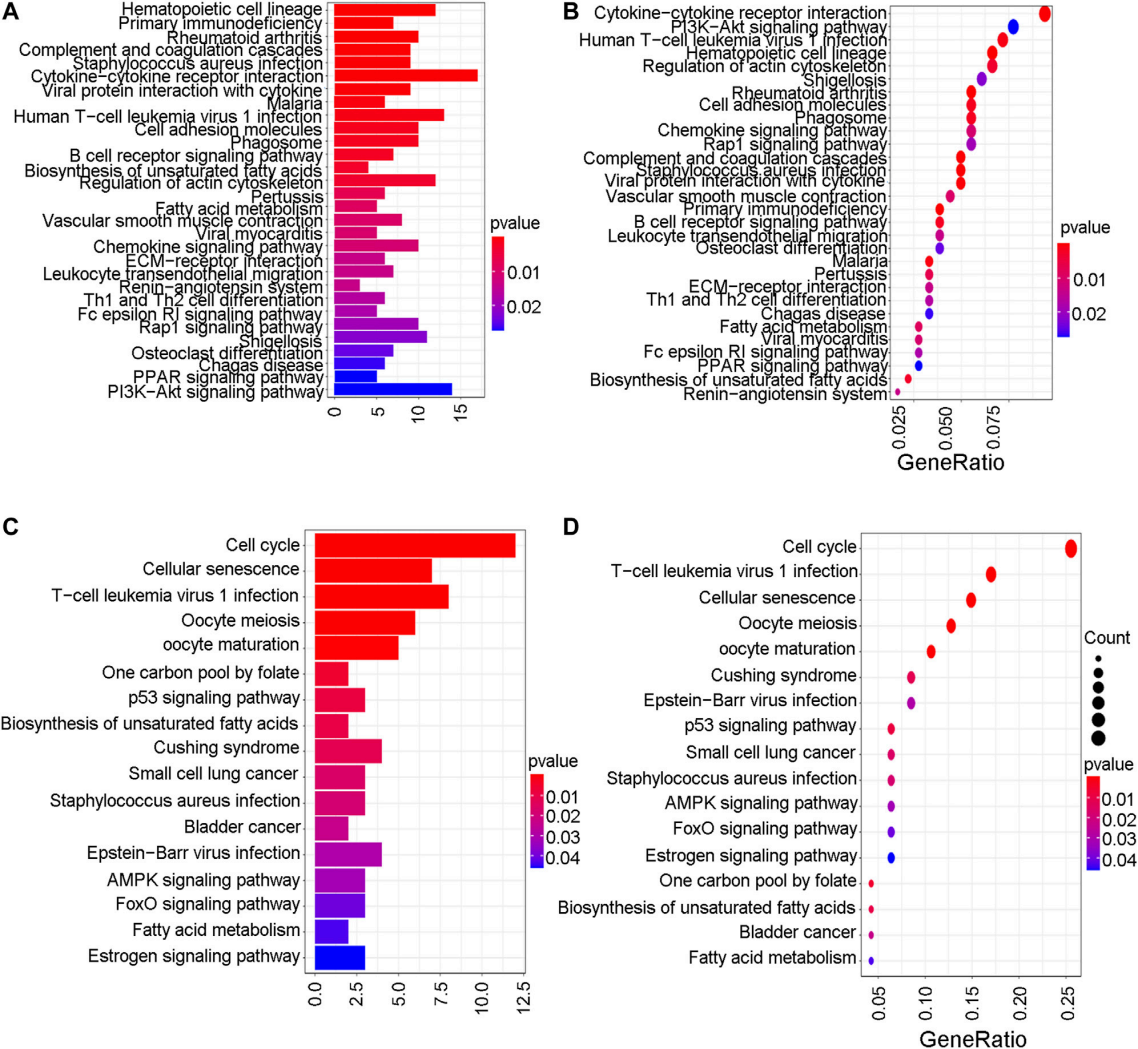
KEGG analysis. **(A)** The KEGG analysis by barplot in TCGA cohort. **(B)** The KEGG analysis by bubble chart in TCGA cohort. **(C)** The KEGG analysis by barplot in GEO cohort. **(D)** The KEGG analysis by bubble chart in GEO cohort.

To further explore the mechanistic pathways or biological functions that were correlated with ferroptosis-related genes during bladder cancer, DEG analysis was performed between the high-risk and low-risk groups. In total, 132 DEGs (Supplementary Table S15) were identified between the high-risk and low-risk groups in the GEO cohort (|logFC| > 1, *P.* adjusted < 0.05). The distribution of DEGs is shown by the gene heatmap (Figure 9A) and volcano map (Figure 9B) (*p* < 0.05). A total of 497 DEGs (Supplementary Table S16) were identified between the high-risk group and the low-risk group in the TCGA cohort (|logFC| > 1, *P.* adjusted < 0.05). All of the above DEGs between the high-risk group and low-risk group were further used to conduct GO enrichment and KEGG pathway analyses. The GO enrichment analysis for the TCGA cohort is illustrated by a bar plot (Figure 10A) and bubble chart (Figure 10B). The GO results for the GEO cohort are also illustrated by a bar plot (Figure 10C) and bubble chart (Figure 10D). Interestingly, many immune-related pathways and oxidase complex reactions were significantly enriched in the present TCGA cohort (adjusted *p* value < 0.05, Figures 10A,B). Many nuclear biological processes or molecular functions were significantly enriched in the GEO cohort (adjusted *p* value < 0.05, Figures 10C,D). KEGG analysis also showed that DEGs in the TCGA cohort risk groups were significantly enriched in various immune-related biological processes, including primary immunodeficiency, cytokine−cytokine receptor interaction, viral protein interaction with cytokine and cytokine receptor, and human T−cell leukemia virus one infection (adjusted *p* value < 0.05, Figures 11A,B). DEGs in the GEO cohort risk groups were significantly enriched in cellular senescence, human T-cell leukemia virus one infection and so on (adjusted *p* value < 0.05, Figures 11C,D). Notably, the KEGG analysis of both the TCGA and GEO cohorts identified various similarly enriched biological processes, including human T-cell leukemia virus one infection, fatty acid metabolism, and biosynthesis of unsaturated fatty acids (Figure 11).

### Single-sample Gene Set Enrichment Analysis

**FIGURE 12:**
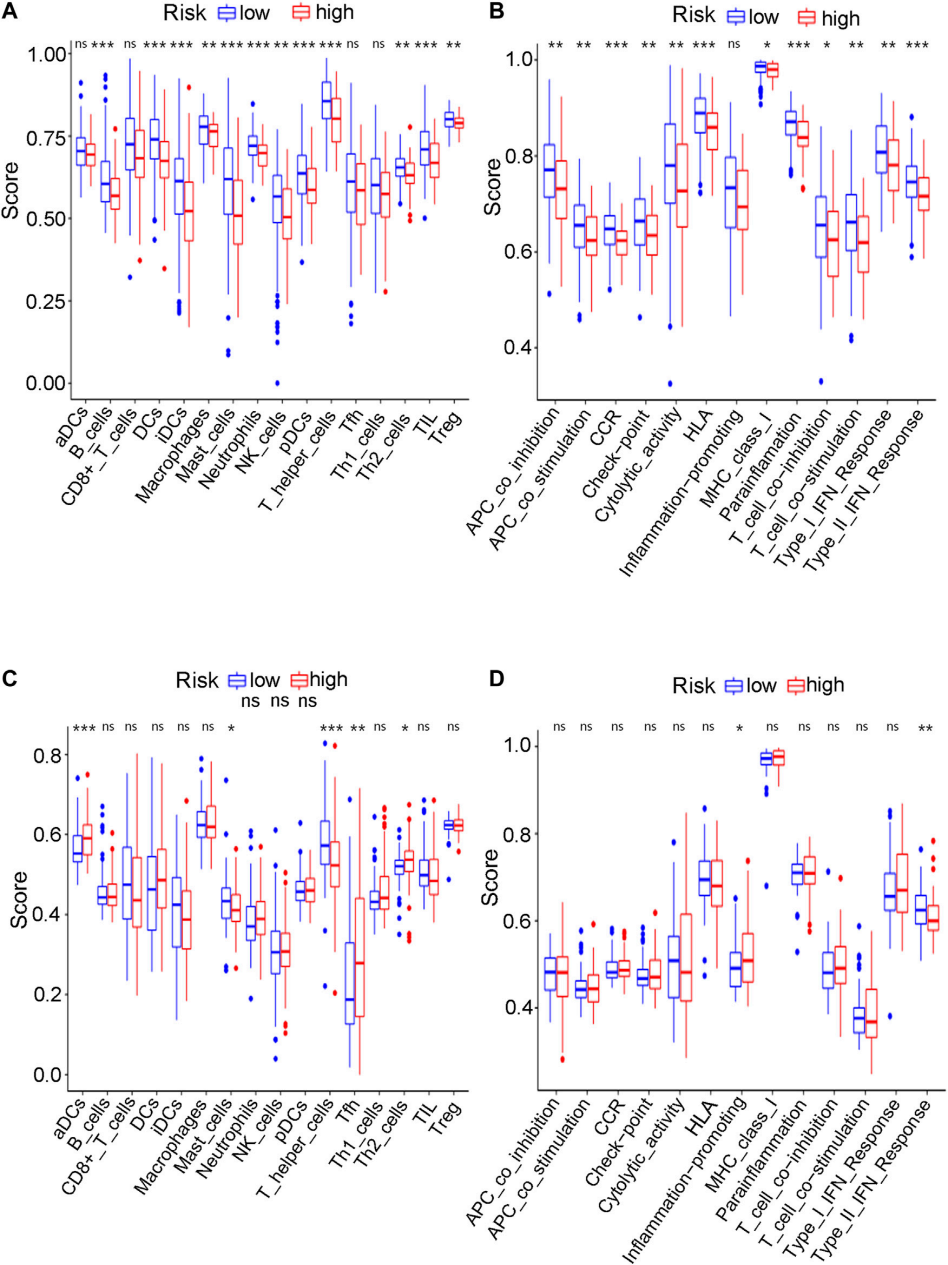
The ssGSEA analysis results. **(A)** The difference of infiltrating score for immune cells in TCGA cohort. **(B)** The difference of infiltrating score for immune-related pathways’ activity in TCGA cohort. **(C)** The difference of infiltrating score for immune cells in GEO cohort. **(D)** The difference of infiltrating score for immune-related pathways’ activity in GEO cohort.

Considering the various enriched immune-related pathways, we further explored the relationship between immune pathways and different risk scores. The enrichment scores of many immune cells and corresponding immune functions and pathways with ssGSEA were quantified both for the GEO cohort (Supplementary Table S17) and the TCGA cohort (Supplementary Table S18) in detail in the present study. Compared to the low-risk group in the TCGA cohort, the higher-risk group obtained lower scores for B cells, DCs, iDCs, mast cells, neutrophils, NK cells, pDCs, T helper cells and Th2 cells (adjusted *p* value < 0.01, Figure 12A). Various antigen presentation processes consisting of APC inhibition, APC stimulation, CCR, checkpoint, T cell inhibition, T cell stimulation, type 1 IFN response and type 2 IFN response were found to be weaker in the high-risk group (adjusted *p* value < 0.01, Figure 12B). Various antigen presentation processes components, including aDCs, mast cells, T helper cells, Th2 cells and type 2 IFN response, were also found to be significantly different in the risk groups (adjusted *p* value < 0.05, Figures 12C,D). Notably, various immune-related cells and biological processes were found to be significantly different in the high-risk group, which is consistent with the results of the above GO enrichment analysis.

## Discussion

Transurethral resection or radical cystectomy is suggested for NMIBC and MIBC, respectively. Early postoperative recurrence and further treatment strategies bring great challenges to current clinical procedures. For metastatic or advanced bladder tumour patients with adequate cardiopulmonary station and renal function (GFR > 50%), a chemotherapy regimen of gemcitabine combined with cisplatin has been given as the first-line treatment. However, various biological pathways, including mismatch repair, nucleotide excision repair and the Fanconi anemia pathway (Torgovnick and Schumacher, 2015), usually enhance DNA damage repair caused by antitumour drugs, further leading to drug resistance. Various mechanistic pathways, including alterations in drug transporters and adduct formation with glutathione, have been reported to reduce the concentration of antineoplastic drugs in the nucleus (Galluzzi et al., 2012). For cisplatin-resistant bladder tumour patients with metastatic or advanced stages, carboplatin-based chemotherapy or an immune checkpoint inhibitor is currently suggested (Nadal and Bellmunt, 2019). However, due to the negative PD-L1 expression or altered gene expression profile of bladder tumours, current immunotherapy treatments hardly obtain complete and lasting antitumour effects. Current chemotherapy and immunotherapy can only give patients short-term therapeutic effects and limited survival benefits. Thus, identifying a new therapeutic mechanism is of great importance for bladder tumour treatment. Ferroptosis is a term for iron-dependent programmed cell death and it was first reported to refer to overactive lipid peroxidation and irregular plasma membrane rupture in 2012 (Dixon et al., 2012). Various recent studies have identified that many advanced cancer cells that are resistant to current conventional chemotherapy or immunotherapy tend to be more sensitive to ferroptosis (Viswanathan et al., 2017; Tsoi et al., 2018; Angeli et al., 2019). These findings undoubtedly bring a new research direction to current cancer treatment. To our knowledge, there have been no reports concerning the relationship between bladder cancer and ferroptosis until now. The present study aimed to identify whether ferroptosis-related genes play a role in bladder tumours and provide a new direction for further scientific research on bladder tumour treatment.

Ferroptosis mainly presents as two biological pathways as follows: iron accumulation and lipid peroxidation. Ferroptosis essentially indicates the imbalance between oxidative damage and antioxidant protection in organisms (Kuang et al., 2020). A network of regulatory mechanisms that are correlated with iron accumulation and lipid metabolism has been reported to induce or inhibit ferroptosis (Fonseca-Nunes et al., 2014). Tumour cells are reported to be more iron-dependent than normal cells for their growth and proliferation (Fonseca-Nunes et al., 2014). This iron-dependent characteristic indicates that many iron regulatory drugs can be used for cancer treatment. Many genes related to lipid peroxidation have also been regarded as future target treatment directions (Yuan et al., 2016; Doll et al., 2017). The analysis of ferroptosis-related genes in bladder tumour patients is of great importance to identify whether bladder tumours may benefit from ferroptosis inducers. The purpose of the present study was to explore the role of ferroptosis-related genes in bladder tumours. The tumour and nontumour differential gene analysis in the present study identified 49 ferroptosis-related genes expressed in both tumour and nontumour samples from either the GEO or TCGA datasets. DEG analysis identified 33 (67.3%) ferroptosis-related genes that were significantly differentially expressed between bladder tumour samples and nontumour samples for the GEO cohort. However, this differentially expressed ferroptosis-related gene proportion for the TCGA cohort was only 51%. The expression files in the GSE13507 series were detected at one institution using one platform, an Illumina human-6 v2.0 expression beadchip (GPL6102) (Kim et al., 2010). The expression files from the TCGA cohorts were derived from different resources. To better explore the possible role of ferroptosis in bladder tumours, we first used the GEO cohort as the training group.

A total of 60 ferroptosis-related genes were identified through previously published studies, and 54 genes (90%) were expressed in the mRNA expression matrix for the present bladder tumour samples. DEG analysis found that approximately 67.3% of ferroptosis-related genes were significantly expressed between bladder tumour tissues and nontumour tissues. These findings suggested that ferroptosis may play an important role in bladder cancer development. Further ferroptosis inducer therapy may become a new treatment direction after chemotherapy and immunotherapy for advanced or metastatic bladder cancer. In total, 54 ferroptosis-related genes were identified in the bladder cancer RNA transcriptome file, and intersection results identified four prognostic DEGs in the present cohort. The subsequent multivariate Cox analysis and survival analysis both validated the predictive value of this ferroptosis gene signature. The validation cohort of the TCGA samples also tested the predictive role of the new gene signature. All statistical results indicated the possible role of ferroptosis in bladder tumour development. The four target genes were ALOX5, FANCD2, HMGCR and FADS2. The Cox survival analysis results showed that ALOX5 was positively correlated with a better prognosis (HR 0.15, *p* = 0.031), and the other three genes, FANCD2, HMGCR and FADS2, were correlated with poor prognosis, with HRs of 1.938, 1.890, and 1.688, respectively (*p* < 0.05).

ALOX5 is a type of lipoxygenase and plays a pivotal role in inducing ferroptosis in various types of cancer cells (Wenzel et al., 2017; Li et al., 2020). Lipid peroxidation has been reported to be the main mechanism of ferroptosis, and this peroxidation mainly includes the peroxidation of polyunsaturated fatty acids (PUFAs). PUFAs mainly contain linoleic acid, docosahexaenoic acids and arachidonic acid (AA). ALOX5 is a metabolic enzyme for AA (Li et al., 2018) and it metabolizes AA into 5-hydroperoxyeicosatetraenoic acid. 5-Hydroperoxyeicosatetraenoic acid is metabolized by glutathione peroxidase into 5-hydroxyeicosatetranoic acid and further metabolized into 5-oxo-eicosatetraenoic acid. ALOX5 has been viewed as a vital regulator of lipid peroxidation, which is currently believed to be the main mechanism of ferroptosis. Excessive accumulation of lipid peroxide contributes to the occurrence of membrane rupture and cell death. Many studies have also identified that the induction of cell death by ALOX5 further promotes the release of damage-associated molecular patterns (Kashyap et al., 2015). The above studies indicate that ALOX5 induces ferroptosis and may be used to help treat cancer patients from a novel perspective. The present study identified ALOX5 as a target gene, further implying that bladder cancer may benefit from novel ferroptosis-related treatments.

FANCD2 (Fanconi anemia complementation group D2) plays a role in DNA damage repair (Nakanishi et al., 2002) and it is a nuclear protein and a negative mediator of the ferroptosis process. When the coding gene of FANCD2 is knocked down, tumour cells are more likely to be damaged by ferroptosis. With the continuous improvement of relevant mechanistic research, FANCD2 has recently been identified to suppress lipid peroxidation and iron metabolism (Song et al., 2016). FANCD2 may participate in autophagy and further play a role in the network between autophagy and ferroptosis (Sertorio et al., 2013). In contrast, FANCD2 may also play a role in ferroptosis through an autophagy-independent mechanism (Song et al., 2016). FANCD2 may prevent erastin-induced GPX4 degradation and reduce tumour sensitivity to ferroptosis. In the present study, the FANCD2 protein was negatively correlated with the expression of ALOX5. High expression of FANCD2 tends to have a worse survival outcome. Survival Cox analysis identified FANCD2 as a risk factor for bladder tumour prognosis in the present study (*p* = 0.021). The heatmap of gene expression for the target genes in Figure 3B intuitively shows that FANCD2 was highly expressed in tumour tissues. Thus, the high expression of FANCD2 in bladder cancer samples allows tumour cells to be less sensitive to ferroptosis to further promote the proliferation and growth of cancer. The above findings indicate that FANCD2 may be a novel treatment target during ferroptosis induction treatment for bladder cancer.

FADS2 (fatty acid desaturase 2) is encoded by genes on chromosome 11 (Noto et al., 2017). FADS2 is an important enzyme that catalyses the metabolism of PUFAs and it plays a role in various diseases, including inflammation, cancers, type 2 diabetes and hypertension. However, there is no unified conclusion about the relationship of FADS2 with the proliferation or growth of cancer cells. Some studies have found that FADS2 expression is significantly lower in tumour tissue than in normal samples. Lower FADS2 expression levels are significantly correlated with a poor prognosis during human breast cancer (Lane et al., 2003). Notably, an increasing number of studies have obtained different opinions and conclusions. Microarray analysis has found that FADS2 expression is significantly upregulated compared to that in normal tissues, especially paracancerous tissues, melanoma tissues, lung cancer tissues and brain cancers (Sun et al., 2006; He et al., 2012; Jiang et al., 2017). FADS2 knockdown leads to a high level of iron accumulation and lipid reactive oxygen species in lung cancer cells, indicating that FADS2 may be a suppressor factor for ferroptosis (Jiang et al., 2017). Jiang also reported that erastin-induced cell death significantly increases when FADS2 is completely deleted. The present study also confirmed the possible role of FADS2 as a suppressor factor for ferroptosis. The high expression level of FADS2 was positively correlated with worse survival prognosis in bladder tumour samples (*p* = 0.036). In the present study, the 4-gene signature was also confirmed to be a risk factor for bladder tumour survival prognosis. Both the GEO cohort and the TCGA cohort in the present study identified the ferroptosis gene signature as an independent risk factor for overall survival.

HMGCR, a 3-hydroxy-3-methyl-glutaryl coenzyme A reductase, plays an important role in the synthesis of mevalonic acid (Shimada et al., 2016). Shimada et al. found that inhibition of HMGCR enhanced FIN-56-induced ferroptosis. HMGCR is upstream of isopentenyl pyrophosphate synthesis, and when it is inhibited by statins, ferroptosis is markedly enhanced. Isopentenyl pyrophosphate is a major metabolite of mevalonate, which is important for CoQ 10 production, the isopentenylation of Sec-tRNA and cholesterol biosynthesis. Abnormal respiratory activity and mitochondrial oxidative damage easily occurs when CoQ 10 production or isopentenyl pyrophosphate synthesis is inhibited (Yang et al., 2016). The present study also identified HMGCR as an inhibitor of ferroptosis (*p* = 0.011), which was consistent with previous studies. Thus, we infer that FADS2, FANCD2 and HMGCR play a negative role in ferroptosis during bladder cancer development. When the expression of HMGCR is upregulated in tumour cells, resistance to cell death is enhanced, further leading to cancer proliferation and metastatic behaviours. Several drugs, including fluvastatin, lovastatin acid and simvastatin, have been identified as target drugs for HMGCR and have a potential anticancer therapy perspective in the future (Shimada et al., 2016; Yang and Stockwell, 2016).

A ferroptosis-related gene signature was built by LASSO Cox regression analysis. Considering the above mechanistic research of each gene, the 4-gene signature in the present study may be theoretically regarded as a valuable grouping tool for risk factors. The high-risk group tended to have a higher proportion of deaths in the GEO cohort (Supplementary Figures S1A and S1C). This different proportion was also validated by the TCGA cohort (Supplementary Figures S1B and S1D). The cut-off value for the TCGA cohort was defined by the median value of the risk score in the GEO cohort. The proportion of samples in the high- and low-risk groups in the TCGA cohort was not balanced (Supplementary Figure S1C), but the proportion of deaths was significantly higher in the high-risk group. The PCA and t-SNE analysis (Figure 4) also showed that the low- and high-risk groups in both the GEO and TCGA cohorts were distributed in completely different directions, indicating the feasibility of the grouping method in the present study. Figure 5 indicates that ferroptosis-related risk scores also play a role in classifying bladder molecular subtypes. The “Neuronal” subtypes tended to obtain the highest risk scores and the “Luminal” subtypes tended to obtain the lowest risk scores (*p* < 0.001) These results were consistent with previous research showing that the neuronal subtypes or high risk scores result in significantly worse survival outcomes. Supplementary Table S12 illustrates that the high-/low-risk groups posed a significant relationship with TP53 alterations. Further basic experimental studies are also needed to explore the TP53-related mechanism of ferroptosis during bladder tumours. Figure 6A illustrates that samples with higher risk scores in the GEO cohort tended to have worse survival prognoses than those in the low-risk group (*p* < 0.05). The predictive value of the present risk group was also validated in the TCGA cohort (*p* < 0.05).

Notably, the stage of bladder tumour was not an independent risk factor for survival analysis in either the GEO cohort or the TCGA cohort. For the GEO cohort, univariate Cox analysis identified age (*p* < 0.001), grade (*p* < 0.001) and risk score (*p* < 0.001) as predictive factors. However, further multivariate Cox analysis identified only age (*p* < 0.01) and risk score (*p* = 0.016) as independent risk factors for patient OS (Figure 7). Because the clinical information for the stage was unavailable during the GEO cohort, the predictive value of the stage could not be explored in our study. However, with complete clinical information, including both the stage and grade of bladder tumours in the TCGA cohort, multivariate Cox regression analysis was performed, and age (*p* = 0.002), stage (*p* < 0.001) and risk score (*p* = 0.006) were identified as independent risk factors for patient OS (Figure 8). The present risk score was derived from the detailed expression of target genes of bladder cancer, and it was theoretically more accurate than the grade or stage in predicting tumour prognosis. Our analysis in both the test cohort and validation cohort confirmed the predictive value of the ferroptosis-related gene signature. Thus, this gene signature can be used as a valuable tool for prognosis in future clinical work.

The mechanism of ferroptosis has become an intense research topic in recent years, but the detailed relationship between ferroptosis and bladder cancer has not been studied. Whether and how ferroptosis plays a role in bladder cancer remain unclear. To further explore the possible basic mechanism, GO enrichment and KEGG analyses revealed that various immune-related biological processes and pathways were enriched. Thus, it can be reasonably assumed that ferroptosis has a close connection with immune-related mechanisms during the development of bladder cancer, which requires basic experimental verification in the future. Wang et al. recently discovered that ferroptosis had a close relationship with the benefit of PD-L1 blockade, indicating that immunotherapies may play roles by promoting ferroptosis in tumour cells (Wang et al., 2019). The KEGG analysis of both the TCGA and GEO cohorts identified various similarly enriched biological processes, including human T-cell leukemia virus one infection, fatty acid metabolism, and the biosynthesis of unsaturated fatty acids. These common biological pathways have recently been reported as the major mechanism of ferroptosis (Wu et al., 2020). Interestingly, the high-risk group in both the GEO and TCGA cohorts tended to have a lower concentration of mast cells and T helper cells (*p* < 0.05). Furthermore, the type II IFN response was found to be significantly weakened in the high-risk group in both the GEO and TCGA cohorts. Thus, we believe that the antitumour immunity for the high-risk samples is impaired, further leading to the growth or metastases of bladder cancer. Many pathway experiments are needed to verify the relationship between ferroptosis and immune-related pathways in the future.

The present study aimed to identify whether ferroptosis-related genes play predictive roles in bladder cancer patients. Therefore, all pathologically confirmed bladder cancer samples were included in the analysis without distinguishing between MIBC and NMIBC. There were 165 bladder cancer samples with available clinical data in the GEO cohort, including 103 (62.4%) NMIBC samples and 62 (37.6%) MIBC samples. For the TCGA cohort, there were only four NMIBC samples (1%) in this study. If only MIBC or NMIBC samples were included in the analysis, the patient numbers would have been too small for reliable statistics. Therefore, larger-scale gene sequencing validation studies on NMIBC alone or MIBC alone are needed in the future. It is worth noting that the risk group in the present study was significantly correlated with MIBC or NMIBC status in both the GEO (*p* = 0.001) and TCGA (*p* < 0.001) cohorts (Table 3).

There are also various limitations during our study. First, our ferroptosis-related gene signature was built and validated by retrospective public data from the GEO and TCGA databases, and further prospective cohort data are needed to validate this model. Second, only ferroptosis-related genes were included in our signature development procedure, and many valuable genes that are not related to ferroptosis may have been excluded from the present study. Furthermore, basic experimental validations are needed to verify the relationship between ferroptosis and bladder cancer.

## Conclusions

We successfully built a ferroptosis-related gene signature to predict the overall survival for bladder cancer patients. This signature has been proven to have significant predictive value in both trial and validation cohorts. This indicates that ferroptosis may play some role in the development of bladder cancer and thus inspires a novel research direction for targeted therapy of bladder cancer in the future.

## Data Availability

The datasets presented in this study can be found in online repositories. The names of the repository/repositories and accession number(s) can be found in the article/Supplementary Material.

## Abbreviations

ALOX5, Arachidonate 5-lipoxygenase; DEGs, Differentially Expressed Genes; ERBB2, erb-b2 receptor tyrosine kinase 2; ERCC2, ERCC excision repair 2; FADS2, Fatty Acid Desaturase 2; FANCD2, Fanconi Anemia Complementation D2; FGFR3, Fibroblast Growth Factor Receptor 3; GEO, Gene Expression Omnibus; GO, Gene Ontology; HMGCR, 3-hydroxy-3-methylglutaryl-CoA Reductase. KEGG, Kyoto Encyclopedia of Genes and Genomes; LASSO, Least Absolute Shrinkage and Selection Operator; RB1, retinoblastoma 1; ssGSEA, Single-sample Gene Set Enrichment Analysis; TCGA, The Cancer Genome Atlas; TP53, Tumor Protein 53
