# Supplementary material for: Construction and External Validation of a Ferroptosis-Related Gene Signature of Predictive Value for the Overall Survival in Bladder Cancer
Source: Front Mol Biosci. 2021 May 21;8:675651. doi: 10.3389/fmolb.2021.675651 (PMC8175978; doi:10.3389/fmolb.2021.675651)
Supplement: Supplementary file 1 [file DataSheet1.ZIP › Supplementary files/Supplementary table12.docx]

Table. Gene mutation data in low and high risk groups

|  | Low risk | High risk | *p* |
| --- | --- | --- | --- |
| TP53 Mutation |  |  |  |
| No, n (%) | 188 (90.4%) | 20 (9.6%) | **0.002** |
| Yes, n (%) | 152 (79.2%) | 40 (20.8%) |  |
| ERBB2 Mutation |  |  |  |
| No, n (%) | 298 (84.7%) | 54 (15.3%) | 0.605 |
| Yes, n (%) | 42 (87.5%) | 6 (12.5%) |  |
| RB1 Mutation |  |  |  |
| No, n (%) | 278 (84.2%) | 52 (15.8%) | 0.357 |
| Yes, n (%) | 62 (88.6%) | 8 (11.4%) |  |
| ERCC2 Mutation |  |  |  |
| No, n (%) | 310 (85.6%) | 52 (14.4%) | 0.272 |
| Yes, n (%) | 30 (78.9%) | 8 (21.1%) |  |
| ATM Mutation |  |  |  |
| No, n (%) | 290 (84.3%) | 54 (15.7%) | 0.333 |
| Yes, n (%) | 50 (89.3%) | 6 (10.7%) |  |

Data presented as number (%) or mean±SD as appropriate. Bold values indicate statistically significant *(p*＜0.05)
