# Supplementary material for: Construction and External Validation of a Ferroptosis-Related Gene Signature of Predictive Value for the Overall Survival in Bladder Cancer
Source: Front Mol Biosci. 2021 May 21;8:675651. doi: 10.3389/fmolb.2021.675651 (PMC8175978; doi:10.3389/fmolb.2021.675651)
Supplement: Supplementary file 1 [file DataSheet1.ZIP › Supplementary files/Supplementary table13.docx]

Table A. Univariate analysis of factors associated with survival status in GEO cohort.

|  | HR (95% CI) | *P* value |
| --- | --- | --- |
| Age | 3.94 (2.24-6.94) | **<0.01** |
| Gender | 0.64 (0.36-1.14) | 0.13 |
| Grade  RiskScore  ALOX5  FANCD2 | 2.74 (1.69-4.43)  2.80 (1.64-4.78)  0.15 (0.03-0.84)  26.03(2.03-333.2) | **<0.01**  **<0.01**  **0.03**  **0.01** |
| HMGCR  FADS2 | 151.9 (2.12-108.3)  36.01 (2.54-510.9) | **0.02**  **<0.01** |

Bold values indicate statistically significant *(p*＜0.05)

Table B. Multivariate analysis of factors associated with survival status in GEO cohort.

|  | HR (95% CI) | *P* value |
| --- | --- | --- |
| Age | 3.79 (2.07-6.95) | **<0.01** |
| Grade  RiskScore  ALOX5  FANCD2 | 1.59 (0.89-2.85)  2.29 (1.27-5.72)  0.23 (0.02-2.58)  0.66 (0.03-17.17) | 0.12  **0.01**  0.24  0.80 |
| HMGCR  FADS2 | 36.34 (0.17-77.05)  14.92 (0.66-35.30) | 0.19  0.08 |
|  |  |  |

Bold values indicate statistically significant. *(p*＜0.05)
