# Supplementary material for: Construction and External Validation of a Ferroptosis-Related Gene Signature of Predictive Value for the Overall Survival in Bladder Cancer
Source: Front Mol Biosci. 2021 May 21;8:675651. doi: 10.3389/fmolb.2021.675651 (PMC8175978; doi:10.3389/fmolb.2021.675651)
Supplement: Supplementary file 1 [file DataSheet1.ZIP › Supplementary files/Supplementary table14.docx]

Table A. Univariate analysis of factors associated with survival status in TCGA cohort.

|  | HR (95% CI) | *P* value |
| --- | --- | --- |
| Age | 1.74 (1.26-2.40) | **<0.01** |
| Gender | 0.86 (0.62-1.20) | 0.38 |
| Grade  RiskScore  ALOX5  FANCD2 | 2.86 (0.71-11.55)  1.05 (1.02-1.08)  0.86 (0.78-0.96)  0.81 (0.64-1.03) | 0.14  **<0.01**  **<0.01**  0.09 |
| HMGCR  FADS2  Stage | 1.23 (0.99-1.54)  1.15 (1.05-1.27)  2.35 (1.61-3.44) | 0.06  **<0.01**  **<0.01** |

Bold values indicate statistically significant *(p*＜0.05)

Table B. Multivariate analysis of factors associated with survival status in TCGA cohort.

|  | HR (95% CI) | *P* value |
| --- | --- | --- |
| Age | 2.04 (1.31-3.19) | **<0.01** |
| Stage  RiskScore  ALOX5  FANCD2 | 2.99 (1.84-4.86)  1.14 (1.07-1.20)  0.86 (0.73-1.02)  0.41 (0.27-0.63) | **<0.01**  **0.01**  0.08  **<0.01** |
| HMGCR  FADS2 | 1.76 (0.87-2.05)  1.15 (1.05-1.27) | 0.13  0.78 |
| Grade | 1.30 (1.06-1.86) | 0.26 |

Bold values indicate statistically significant. *(p*＜0.05)
