# Supplementary material for: Construction and External Validation of a Ferroptosis-Related Gene Signature of Predictive Value for the Overall Survival in Bladder Cancer
Source: Front Mol Biosci. 2021 May 21;8:675651. doi: 10.3389/fmolb.2021.675651 (PMC8175978; doi:10.3389/fmolb.2021.675651)
Supplement: Supplementary file 1 [file DataSheet1.ZIP › Supplementary files/Supplementary table5.docx]

| Pearson correlation analysis | | | | | |
| --- | --- | --- | --- | --- | --- |
|  | | ALOX5 | FANCD2 | HMGCR | FADS2 |
| ALOX5 | Coefficient | 1 | -.022 | .036 | -.359^**^ |
|  | *p* |  | .783 | .649 | .000 |
|  | N | 165 | 165 | 165 | 165 |
| FANCD2 | Coefficient | -.022 | 1 | .440^**^ | .362^**^ |
|  | *p* | .783 |  | .000 | .000 |
|  | N | 165 | 165 | 165 | 165 |
| HMGCR | Coefficient | .036 | .440^**^ | 1 | .365^**^ |
|  | *p* | .649 | .000 |  | .000 |
|  | N | 165 | 165 | 165 | 165 |
| FADS2 | Coefficient | -.359^**^ | .362^**^ | .365^**^ | 1 |
|  | *p* | .000 | .000 | .000 |  |
|  | N | 165 | 165 | 165 | 165 |
| ** *P* < 0.01 | | | | | |
