# Supplementary figures and images for: Construction and External Validation of a Ferroptosis-Related Gene Signature of Predictive Value for the Overall Survival in Bladder Cancer
Source: Front Mol Biosci. 2021 May 21;8:675651. doi: 10.3389/fmolb.2021.675651 (PMC8175978; doi:10.3389/fmolb.2021.675651)

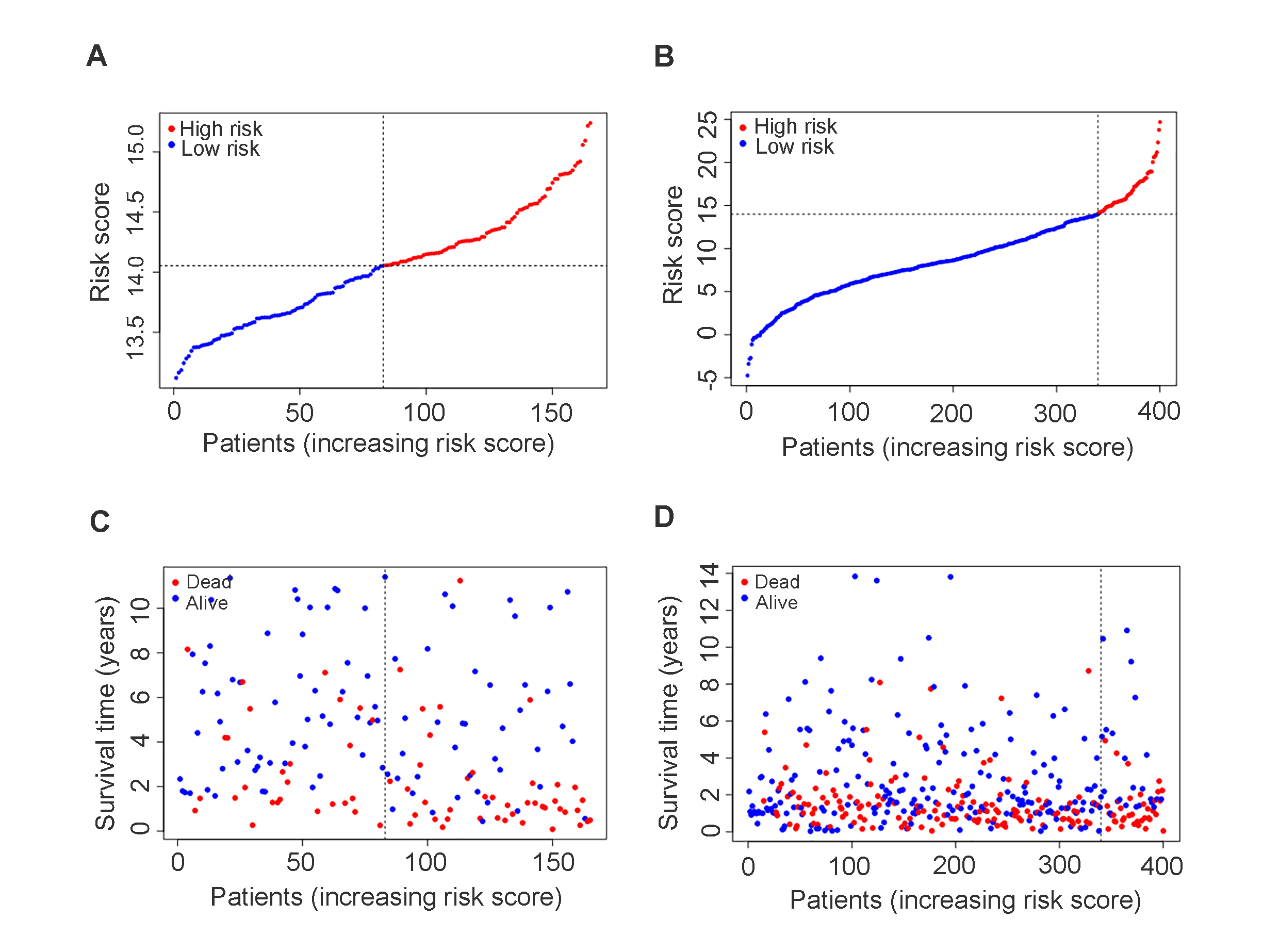

Supplement: Supplementary file 1 [file DataSheet1.ZIP › Supplementary files/Supplementary Figure 1.jpg]
